# Subcellular Partitioning of Trace Elements Is Related to Metal Ecotoxicological Classes in Livers of Fish (*Esox lucius; Coregonus clupeaformis*) from the Yellowknife Area (Northwest Territories, Canada)

**DOI:** 10.3390/toxics13050410

**Published:** 2025-05-19

**Authors:** Aymeric Rolland, Mike Palmer, John Chételat, Marc Amyot, Maikel Rosabal

**Affiliations:** 1 Groupe de Recherche Interuniversitaire en Limnologie (GRIL), Département des Sciences Biologiques, Université du Québec à Montréal (UQAM), Montréal, QC H2X 1Y4, Canada; aymeric.rolland@outlook.com; 2 North Slave Research Centre, Aurora Research Institute, Aurora College, Yellowknife, NT X1A 2R3, Canada; mpalmer@auroracollege.ca; 3 Environment and Climate Change Canada, National Wildlife Research Centre, Ottawa, ON K1S 5B6, Canada; john.chetelat@ec.gc.ca; 4 Groupe de Recherche Interuniversitaire en Limnologie (GRIL), Département de Sciences Biologiques, Université de Montréal, Montréal, QC H2V 0B3, Canada; m.amyot@umontreal.ca

**Keywords:** detoxification, toxicity, metal-handling strategies, fish, quantitative ion character-activity relationships, metal-binding properties

## Abstract

The subcellular partitioning of trace elements (TEs) may depend on their binding preferences, although few field data are available from mining-impacted areas. Northern pike and lake whitefish were collected from different aquatic systems located in the Yellowknife mining area (Northwest Territories, Canada) to examine the subcellular partitioning of TEs in liver cells. Elements belonging to metal classes based on binding affinities were considered: A (Ce, La), borderline (As, Pb), and class B (Ag, Cd). Measurements in the metal-detoxified fractions (granule-like structures and heat-stable proteins and peptides) and in the putative metal-sensitive fractions (heat-denatured proteins, mitochondria and microsomes, and lysosomes) revealed marked differences among metal classes. In both fish species, Cd and Ag accumulated more as detoxified forms (higher than 50%, likely bound to metallothionein-like proteins) than La and Ce (not more than 20%). The two borderline TEs (As and Pb) showed an intermediate behavior between classes A and B. Similar proportions were found in the “sensitive” subcellular fractions for all TEs, where quantitative ion character-activity relationships (QICARs) indicated the covalent index and electronegativity as predictors of the TE contribution in this compartment. This study supports the use of classes of metals to predict the toxicological risk of data-poor metals in mining areas.

## 1. Introduction

The prediction of biochemical interactions of trace elements in biological systems has often been addressed by relating the physical or chemical properties of these contaminants with toxicological parameters [1,2,3]. To distinguish the wide range of potential metal interactions within living cells, some pioneer works [4,5] have proposed a classification of these TEs into three classes (class A, class B, and borderline) based on their binding preferences for O-, N-, or S-containing biomolecules. Elements of class A (e.g., Al^+3^, Ca^+2^, Ce^+3^, La^+3^, Mg^+2^), which are highly stable and known as hard ions, bind anions with oxygen as electron donors, whereas class B elements (e.g., Ag^+1^, Cd^+2^, Hg^+2^, Pb^+2^, Tl^+1^) exhibit a pronounced preference for sulfur and nitrogen donors available on intracellular biomolecules. Borderline elements (e.g., As^+3^, Cu^+2^, Zn^+2^, Pb^+2^, Sb^+3^) are expected to show ligand-binding characteristics that are intermediate between those of classes A and B. Other modifications of this initial classification have been recently proposed to better understand metal interactions with aquatic organisms [3,6,7]. In spite of some successful efforts [1,7,8,9], a generalized prediction of metal toxicity based on chemical characteristics has not yet been achieved. Metal toxicity is a consequence of the wide range of metal interactions within biological systems where the chemical properties of metals play an important role, along with the reactivity of the metals with intracellular compounds and the ability to be chelated with or bound to biomolecules involved in detoxification as well as the stability of such complexes [8,9]. In addition, the nature of the biomolecules targeted by these contaminants and the effects of metal on the biological function of these biomolecules are also factors to be considered, as they can result in deleterious effects [7,10].

One promising way to further our understanding of metal toxicity is through the examination of subcellular metal partitioning in metal-contaminated organisms, which has proven to be a useful tool to evaluate the likelihood of toxic effects following metal bioaccumulation [11,12,13]. This approach allows researchers to distinguish TE burdens between biologically “detoxified” forms (found in the cytosolic heat-stable proteins and peptides (HSP) and in the granule-like fractions) and physiologically sensitive targets (cytosolic enzymes, receptors, or carriers grouped in the heat-denatured fractions (HDP)) and organelles (e.g., mitochondria and microsomes and lysosomes fractions), where metal binding can induce deleterious effects. By doing so, subcellular metal measurements in aquatic organisms can also reveal the metal-handling strategies used by aquatic organisms to cope with these contaminants [14,15,16,17,18]. In addition to identifying subcellular targets of accumulated TEs, these measurements help to understand how some organisms are more tolerant or sensitive to highly contaminated environments, as well as how some elements induce greater damage than others [17,19,20,21,22]. In addition, previous studies have revealed differences in the subcellular partitioning of TEs belonging to different classes in field-collected organisms [17,23], highlighting the need for more understanding regarding the TE behaviors based on quantitative ion character-activity relationships (QICARs) [1].

Aquatic organisms contaminated by a suite of TEs with contrasting chemical behaviors offer the opportunity to examine the potential toxicity of metal accumulated in subcellular fractions. In this regard, fish species inhabiting the Yellowknife area (Northwest Territories, NWT, Canada), which have been chronically exposed to various TEs (mainly arsenic) released from historical gold mining activities, are suitable to study such issues [24]. The legacy contamination developed in the Yellowknife area for more than 50 years is not only restricted to As and Pb but also to other trace metals, such as Ag, Cd, Pb, and Cu [24]. Mining contamination is of local concern since freshwater resources in the region have been used for harvesting food sources and for recreation, particularly for local Indigenous communities, and elevated TE concentrations in fish tissues have been reported to be associated with periods of higher mining emissions [24]. Recent research conducted on various fish species, including lake whitefish (*Coregonus clupeaformis*) and northern pike (*Esox lucius*) from Yellowknife Bay, has observed an important decrease in As concentrations in the liver cells of these fish compared to those reported in the 90s [24]. However, the TE levels measured in *C. clupeaformis* and *E. lucius* still show signs of metal contamination, potentially leading to deleterious effects.

In view of this information, our goal was to determine the concentrations of several TEs (Ag, As, Cd, Pb), including some rare earth elements (REEs; Ce, La), in lake whitefish and northern pike in isolated subcellular fractions and to assess the potential toxicity of these contaminants based on metal partitioning. In addition, comparisons between the metal-handling strategies used for the different metal classes provided insights into the biochemical interactions of TEs within biological systems. QICAR regression models considering the TE contributions in the subcellular fractions as well as in both compartments were explored for further analyses regarding metal behavior at the subcellular level. We anticipated that differences in the subcellular partitioning of such TEs, which differ in their biochemical behaviors and affinity for chemical groups (S, N, O), would be observed. We hypothesized that Class B metals would be found in higher proportions as detoxified forms than Class A metals and borderline elements would show subcellular distributions as Class A and B metals have. We also expected to identify some chemical properties or indexes related to measured TEs as predictors of their accumulation within the cells.

## 2. Materials and Methods

### 2.1. Sampling Sites

Four sites located in the mining-impacted Yellowknife area were chosen based on previously reported TE concentrations in water, sediments, and biota [25,26,27] (Supplementary Figure S1). These sites exist along a gradient of contamination, representing differences in total concentrations for Ag, As, Cd, and Pb in different environmental matrices. In Yellowknife Bay, which has been chronically contaminated by mining activities in the area since the 1940s [28], two sites were sampled. The Bay 1 site (62°29.223′ N, 114°20.227′ W) is located close to the outlet of the Baker Creek effluent of the Giant Mine. The Bay 2 site (62°17.979′ N, 114°21.067′ W) is 20 km south, at the entry of Yellowknife Bay. Both sites have been sampled in previous studies, revealing contrasting total TE (As, Cd) concentrations in various fish species [29,30,31]. The Long Lake sampling site (62°47.510′ N, 114°43.037′ W), located downwind of the roasting site at the Giant Mine, also received emissions from mining operations. In this sampling site, TE data in sediments and biota have been associated with these anthropogenic sources [32]. The Small Lake site (62°52.098′ N, 113°82.909′ W) is located 30 km upwind from the roaster of the Giant Mine [33], preventing significant contaminant input by Yellowknife mining activities. In addition to the TEs associated with mining operations in this area (Ag, As, Cd, Pb), our measurements also focused on REEs, which are expected to increase in aquatic environments due to their growing application in clean technology [34]. The first REE mining operation in Canada recently started its activities in the Yellowknife area, and background environmental concentrations for these contaminants are needed [35]. In this work, six TEs of different metal classes were studied: Class B (Ag and Cd) and Class B (La and Ce) [5]. With regard to As and Pb, we applied the classification done by Nieboer and Richardson [4], where As^+3^ and Pb^+3^ were considered as borderline elements.

### 2.2. Fish Collection

Northern pike (NP, *Esox lucius*) and lake whitefish (WF, *Coregonus clupeaformis*) were selected for our study because of their wide distribution in the Yellowknife area. Both fish species are also easy to collect and abundant in the study lakes. Northern pike and lake whitefish feed at different trophic levels [36]. Northern pike are piscivorous as adults, and cannibalism is also common in Arctic lakes [37]; therefore, they have a higher trophic position (inferred from nitrogen stable isotope [δ^15^N] measurements) than lake whitefish. The latter species is a benthivore and feeds mostly on mollusks, amphipods, and insect larvae [38,39,40]. In Yellowknife Bay, carbon stable isotope (δ^13^C) measurements in dorsal muscle from these species indicated NP and WF were primarily littoral feeders [30]. At each sampling site, fish were collected using gill nets. Fish were collected in 2015 from the two Yellowknife Bay sites (August), from Long Lake (in July 2018), and from Small Lake (in January 2019). At both Yellowknife Bay sites, six fish of both species were captured, while eight fish of both species were captured at Small Lake, and, at Long Lake, five northern pike and ten lake whitefish were taken. The liver was the selected target organ because of its importance in detoxification processes. Livers were dissected, placed in acid-washed cryovials, and flash-frozen using liquid nitrogen (−196 °C). Samples were kept at −80 °C until analysis. Fish ages were estimated by AAE Tech Services Inc. (Manitoba, Canada) using collected otoliths [41]. Muscle tissues were also collected for carbon- and nitrogen-stable isotope analyses performed by the light-stable isotope geochemistry laboratory of Geotop (UQAM, Canada). Capture and sampling protocols for both species were approved by the UQAM Animal Care Committee (No. 0518-946-0519) and according to the Northwest Territories Scientific Research Licence (No. 16207).

### 2.3. Subcellular Partitioning of Trace Elements in Liver Cells

As protocols for the subcellular metal partitioning are species-specific [12,35], and protocols have not been adapted for liver cells of NP and WF, enzymatic validation was required before application. The efficiency of the protocol was assessed by enzymatic bioassays applied to subcellular fractions to determine the distribution of three specific marker enzymes [42,43,44] (Supplementary Figure S2). The application of this approach was performed in 2019. The following fractions were isolated: debris and nuclei; NaOH-resistant (referred to as granule fraction); mitochondria; microsomes and lysosomes; heat-stable proteins and peptides (HSP), including metallothionein (MT) or metallothionein-like proteins and peptides (MTLP); and heat-denatured proteins (HDP) (Supplementary Figure S3). Putative metal-sensitive compartments (MSC) account for TE accumulation in the mitochondria, the microsomes and lysosomes, and the heat-denatured (HDP) fractions, while metal-detoxified compartments (MDC) include TE content in the granule as well as in the heat-stable proteins and peptides (HSP) fractions.

For each fish, a liver sample of around 130 mg was chopped using a surgical steel razor blade and placed in a Teflon tube with homogenization buffer containing 25 mM Tris (Sigma-Aldrich, Oakville, ON, Canada) and 250 mM sucrose (Sigma-Aldrich, Oakville, ON, Canada) at a pH of 7.4 at a proportion of 1:4.5 (liver weights in mg: buffer volume in µL). Each sample was first homogenized using a Potter-Elvehjem pestle (equipped with a Teflon pestle) five times for 2 s, each time with 30 s of breaks. This homogenate was centrifuged at 1500 *g* for 15 min at 4 °C. After centrifugation, the supernatant was separated, and the pellet was resuspended with the same homogenization buffer at a ratio of 1:4.5 to homogenize a second time using an ultrasonic probe at 20 W, 0.2 s/s for 30 s. This second homogenate was pooled with the first supernatant (final dilution 1:9), mixed with a vortex, and a 100 µL aliquot was collected for mass balance calculations. The resulting homogenate was centrifuged at 1500 *g* for 15 min at 4 °C; the supernatant (S1) was separated for the subsequent centrifugation steps, and the pellet (P1) was digested by adding 500 µL of ultrapure water and heating at 95 °C for 2 min, then adding 500 µL of sodium hydroxide (1 M, ACS grade, Sigma-Aldrich, Oakville, ON, Canada) and heating at 60 °C for 60 min. The subsequent digestate was centrifuged at 10,000 *g* for 10 min at 20 °C, and the supernatant (S2) contained the debris and nuclei fraction, while the pellet (P2) contained the granule fraction. Concurrently, the supernatant (S1) was centrifuged at 25,000 *g* for 30 min at 4 °C, the pellet (P3) was collected as the mitochondria fraction, and the supernatant (S3) was transferred to a new microtube to be ultracentrifuged at 190,000 *g* for 60 min at 4 °C to separate the supernatant (S4) from the microsomes and lysosomes fraction (P4). The supernatant (S4) was heat shocked by placing samples at 80 °C for 10 min and then cooled on ice for 60 min. The resulting sample was centrifuged at 50,000 *g* for 10 min at 4 °C, separating the heat-stable proteins and peptides fraction (HSP, S5) from the heat-denatured proteins (HDP, P5). Ultracentrifugation steps (25,000 *g*, 50,000 *g*, and 190,000 *g*) were carried out using a Beckman Optima LE-80K centrifuge (Beckman Counter, Montreal, QC, Canada) with a SW-55Ti rotor (Beckman Counter, Montreal, QC, Canada), and for the other centrifugation steps, a Sorvall™ Legend™ Micro 21R Microcentrifuge (Thermo Fisher Scientific, Mississauga, ON, Canada) was used.

### 2.4. Trace Element Measurements and Quality Controls

For all TE analyses, the laboratory ware was soaked in 15% nitric acid. All samples were freeze-dried (Labconco, Guelph, ON, Canada). For the total TE concentration in the liver, 10 mg (dry weight, dw) was digested overnight with 1 mL of HNO_3_ (OmniTrace Ultra™, MilliporeSigma, Thermo Fisher Scientific, Mississauga, ON, Canada) in a Teflon vial at 60 °C. Another 270 µL of HNO_3_ (OmniTrace Ultra™, MilliporeSigma, Thermo Fisher Scientific, Mississauga, ON, Canada) was then added, and the sample was placed for 3 h in an industrial pressure cooker (121 °C, 15 psi). Samples were then placed overnight at room temperature with the addition of 150 µL HCl (OmniTrace Ultra™, MilliporeSigma, Thermo Fisher Scientific, Mississauga, ON, Canada) and 250 µL H_2_O_2_ (Optima grade, Thermo Fisher Scientific, Mississauga, ON, Canada). The final digestate was placed in a 15 mL metal-free tube (VWR) and completed to 15 mL with Milli-Q water before analysis [44]. For all subcellular fractions, samples were submitted under a digestion protocol to enable TE detection by dissolving any biological tissues and residues. To do so, each fraction was digested using 250 µL HNO_3_ (Optima grade, Thermo Fisher Scientific, Mississauga, ON, Canada) at room temperature for 24 h, then heated at 60 °C overnight. Hydrogen peroxide (250 µL, Optima grade; Thermo Fisher Scientific, Mississauga, ON, Canada) was added to samples containing non-digested residues for 24 h at room temperature. Ultra-pure water was added to reach 2% HNO_3_ (*v*/*v*) for TE measurements [43].

All TE measurements were performed using an ICP-MS/MS (Agilent 8900 Triple Quadrupole ICP-MS, Agilent Technologies, Santa Clara, CA, USA). To ensure the quality and accuracy of the data, we processed blanks and certified reference materials (CRM) concurrently with our samples. Non contamination was observed in blank samples, and the recovery means obtained were between 81% and 126% (Supplementary Table S1). The homogenate aliquot taken before the first centrifugation step was analyzed alongside the other samples for mass balance calculations. From this aliquot, we estimated the total intracellular TE burden and compared it to the total sum of TE burdens in each fraction to assess possible loss or contamination during the fractionation procedure. This mass balance control was assessed using a recovery ratio defined as the total sum of TE burden considering each fraction divided by the total intracellular TE burden estimated from the homogenate (aliquot taken before the first centrifugation step), multiplied by 100 to give this recovery ratio as a percentage (%). We applied our subcellular partitioning protocol to 25 livers of NP and 30 livers of WF. Because there was great variability in the recovery percentages, we decided to only include individuals for which the recovery was between 60–140% in both fish species [23,43]. The final mass balance recovery means varied from 84% to 108% (Supplementary Table S2).

### 2.5. Calculations and Statistical Analysis

Kruskal-Wallis tests followed by Dunn’s tests using Bonferroni correction were applied to determine differences in total hepatic TE concentrations among sites, in TE concentrations or relative contributions among the subcellular fractions (e.g., mitochondria, HSP), and in each compartment among metals. Comparison of the TE concentration or contribution between the two compartments (MSC: metal-sensitive compartment; MDC: metal-detoxified compartment) was also determined using the Wilcoxon-Mann-Whitney test. Relationships between TE contributions and the index or chemical properties (e.g., electronegativity, ionization potential, covalent index) of measured metals were initially examined using bivariate scatterplots for each subcellular fraction and toxicological compartment. When we observed potential relationships between these variables, regression models were assessed and statistical variables were reported (e.g., R^2^, *p*). The assumptions of normality and homogeneity of variance were tested using the Shapiro-Wilk and Levene’s tests, respectively.

## 3. Results

### 3.1. Total Trace Element Concentrations

**Figure 1:**
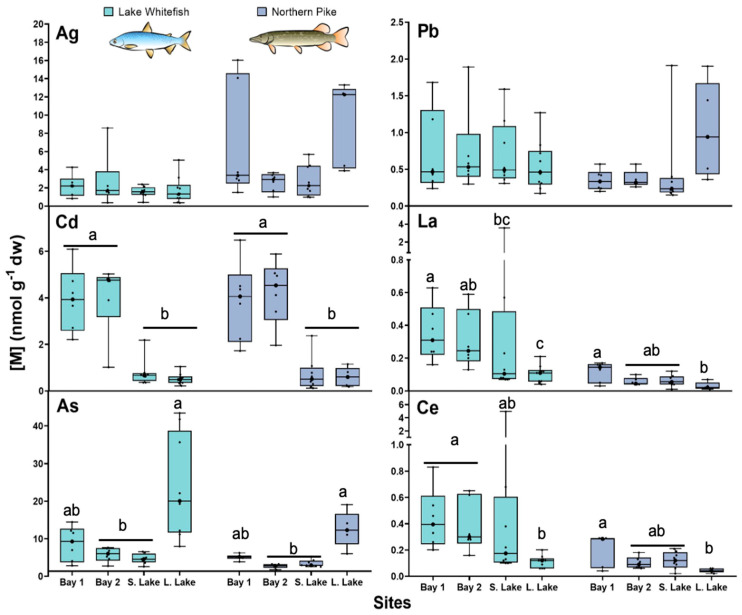
Box and whisker distribution of total concentrations (means ± SD, nmol g^-1^ dw) of Ag, Cd, As, Pb, La, and Ce in the liver of lake whitefish (*Coregonus clupeaformis*; n = 6–10) and northern pike (*Esox lucius*; n = 5–8) collected from the Yellowknife area. Each dot represents an individual fish value. Bars with different letters indicate that the differences are significant (Kruskal-Wallis test followed by Dunn’s test using Bonferroni correction, *p* < 0.05).

Total TE concentrations in the livers of both species showed significant spatial differences among the sampled sites (Figure 1). Both NP and WF collected from Long Lake were the most contaminated in As (Figure 1). For the other TEs examined, both fish species from Bay 1 and Bay 2 sites on Yellowknife Bay had a higher hepatic mean concentration of Cd than those collected from Small Lake and Long Lake (Figure 1). Fish captured from the Bay sites (mainly from Bay 1) generally had higher concentrations of REEs than those from the smaller inland lakes (Figure 1). No significant difference between Small Lake and other sites was observed in terms of Ag and Pb, but fish from this site showed the lowest bioaccumulation values for the studied TEs. Concentrations of TEs in WF liver were collectively greater than those observed for NP. Total TE concentration gradients (calculated as a ratio between the most and the least contaminated fish) varied from 14 (La) to 54 (Cd) and from 12 (Ag) to 27 (Cd) for WF and NP, respectively (Supplementary Table S3). The ranges (minimum-maximum values) of total length, body weight, and age were similar for both fish species.

### 3.2. Subcellular Metal Partitioning

**Figure 2:**
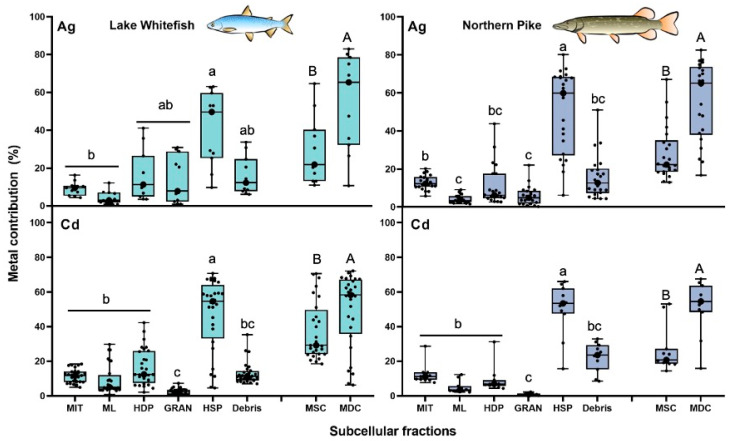
Box and whisker distribution of the contribution (%, n = 10–28) of Ag (**upper panels**) and Cd (**lower panels**) in each subcellular fraction and compartment of the liver of lake whitefish (*Coregonus clupeaformis)* and northern pike (*Esox lucius*). Each dot represents an individual fish value. Bars with different letters indicate that the differences are significant (lowercase letters for subcellular fractions: Kruskal-Wallis test followed by Dunn’s test using Bonferroni correction; capitalized letters for compartments: Wilcoxon-Mann-Whitney test, *p* < 0.05). MIT: mitochondria; ML: microsomes and lysosomes; HDP: heat-denatured proteins; GRAN: granules; HSP: heat-stable proteins and peptides; MSC: metal-sensitive compartment; MDC: metal-detoxified compartment.

**Figure 3:**
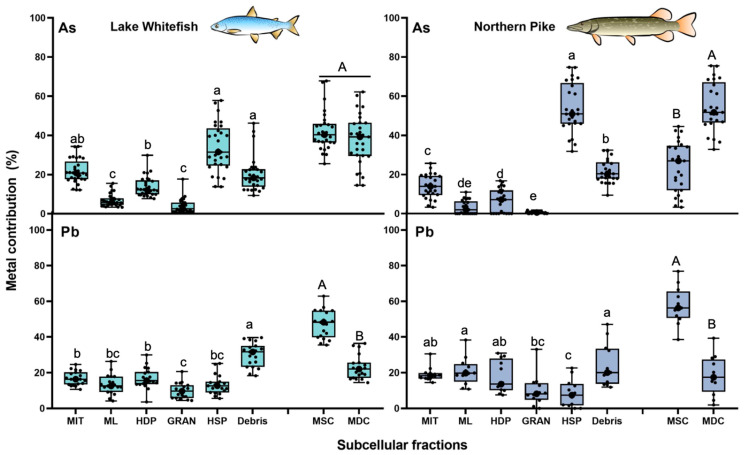
Box and whisker distribution of the contribution (%, n = 19–29) of As (**upper panels**) and Pb (**lower panels**) in each subcellular fraction and compartment of the liver of lake whitefish (*Coregonus clupeaformis)* and northern pike (*Esox lucius*). Each dot represents an individual fish value. Bars with different letters indicate that the differences are significant (lowercase letters for subcellular fractions: Kruskal-Wallis test followed by Dunn’s test using Bonferroni correction; capitalized letters for compartments: Wilcoxon-Mann-Whitney test, *p* < 0.05). MIT: mitochondria; ML: microsomes and lysosomes; HDP: heat-denatured proteins; GRAN: granules; HSP: heat-stable proteins and peptides; MSC: metal-sensitive compartment; MDC: metal-detoxified compartment.

**Figure 4:**
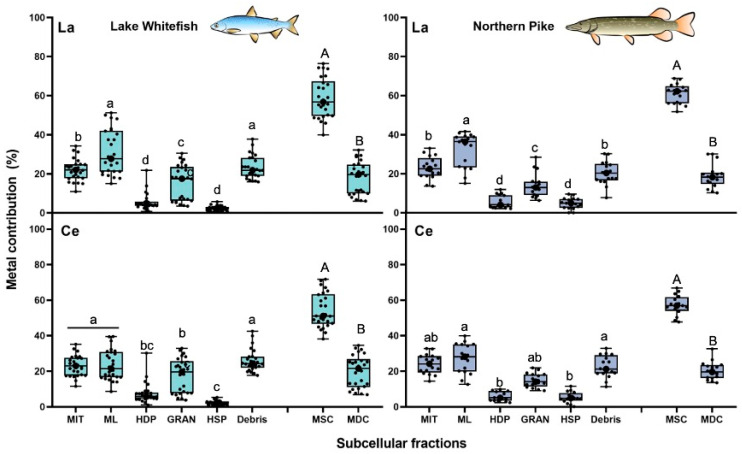
Box and whisker distribution of the contribution (%; n = 16–26) of La (**upper panels**) and Ce (**lower panels**) in each subcellular fraction and compartment of the liver of lake whitefish (*Coregonus clupeaformis)* and northern pike (*Esox lucius*). Each dot represents an individual fish value. Bars with different letters indicate that the differences are significant (lowercase letters for subcellular fractions: Kruskal-Wallis test followed by Dunn’s test using Bonferroni correction; capitalized letters for compartments: Wilcoxon-Mann-Whitney test, *p* < 0.05). MIT: mitochondria; ML: microsomes and lysosomes; HDP: heat-denatured proteins; GRAN: granules; HSP: heat-stable proteins and peptides; MSC: metal-sensitive compartment; MDC: metal-detoxified compartment.

To provide insights into the subcellular partitioning of TEs belonging to different classes, relative contributions (Figure 2, Figure 3 and Figure 4) and TE concentrations (in %, Supplementary Figures S5–S7) were compared among the subcellular fractions (e.g., mitochondria, granules) and between both toxicological compartments (metal-sensitive and detoxified-metal compartments). In this work, we did not include the TE content of the debris fraction given its ambiguous nature, as recommended previously [22,43]. Therefore, the total sum of TE contributions of both compartments was not necessarily up to 100%.

#### 3.2.1. Silver and Cadmium

In proportional terms, the HSP fraction contributed the most to the total intracellular Ag burden in NP (average of 50 ± 22%) and in WF (average of 47 ± 20%) liver cells (Figure 2). Similar metal contributions to those reported for Ag were also obtained for Cd in the HSP fraction isolated from NP (50 ± 15%) and WF (47 ± 20%) livers, which were significantly higher than the percentages found in the other subcellular fractions (Figure 2). For both TEs, lower contributions were reported for granules as well as for the microsomes and lysosomes fractions in NP (ranging from 1% to 6%) and WF (varying from 3% to 14%) cells. Consequently, more than half of the intracellular Ag and Cd was consistently found in the metal-detoxified compartment in WF (Ag: 57% ± 26%; Cd: 50% ± 21%) and NP (Ag: 56% ± 20%; Cd: 51% ± 15%) cells (Figure 2). In contrast to the higher TE proportions in metal-detoxified compartments, the contributions of the putative metal-sensitive compartments to the total intracellular Ag burden were 28% ± 14% and 29% ± 17% for NP and WF, respectively. For Cd, these percentages varied from 27% ± 13% (for NP) to 37% ± 17% (for WF). The average proportions of such detoxified forms of both class B metals were significantly higher than the contributions observed for the metal-sensitive compartment (*p* < 0.001) (Figure 2).

For both class B elements, the concentration measured in the HSP fraction of WF and of NP cells was significantly higher than the average TE content reported for the other subcellular fractions (Supplementary Figure S4). The Cd concentration in the subcellular fractions isolated from both fish species consistently followed the decreasing order (without considering the debris fraction): HSP > mitochondria, microsomes and lysosomes, HDP > granules. For Ag, this order in NP cells was: HSP > mitochondria > HDP > microsomes and lysosomes, granules, and in WF cells it followed: HSP~granules, HDP > mitochondria, microsomes and lysosomes (Supplementary Figure S4). In the HSP fraction, the mean concentration of Ag (1.3 ± 1.1 nmol g^−1^) and Cd (2.0 ± 1.3 nmol g^−1^) in NP cells resulted in 3.3-fold and 4.0-fold higher TE levels than metal levels found in the second more-contaminated fractions, respectively. For WF cells, the average concentration in this cytosolic fraction for Ag (0.63 ± 0.55 nmol g^−1^) and for Cd (0.71 ± 0.61 nmol g^−1^) was also much higher (from 2.7 to 2.9 times) than the level measured in the fraction of the second greater TE accumulation (Supplementary Figure S4).

#### 3.2.2. Arsenic and Lead

Similar to Cd and Ag, the total intracellular As in NP cells was largely associated with the HSP fraction (54% ± 13%) (Figure 3). In WF cells, the mean percentage did not exceed 50% (34% ± 13%), but it was higher than the mean values reported for the other fractions (Figure 3). For Pb, there were no significant differences in the TE contribution among the subcellular fractions (Figure 3). Comparing proportions between both compartments, As was more accumulated in the metal-detoxified compartment (54% ± 12%) of NP liver cells than in the “sensitive” sites (24% ± 13%). In contrast to NP, no difference was observed in the As contribution between both compartments (sensitive sites: 42% ± 10%; detoxification compartment: 38% ± 13%) in WF liver. For both animal models, Pb accumulation in the metal-sensitive sites (WF: 46% ± 8%; NP: 58% ± 11%) was consistently higher than the combined contribution of the HSP and the granule fractions (WF: 23% ± 7%; NP: 18% ± 11%) (Figure 3).

With regards to TE concentrations, As content in the HSP fraction was consistently higher than those measured in the other subcellular fractions for both fish species (Supplementary Figure S5). This cytosolic fraction of NP (2.4 ± 1.9 nmol g^−1^) and WF (3.4 ± 2.2 nmol g^−1^) cells accumulated more than twice the As than the mitochondria fraction (the second most contaminated fraction). In general, low As concentrations were reported in the granule and the microsomes and lysosomes fractions of both fish liver (Supplementary Figure S5). The concentration of As in the subcellular fractions followed the decreasing order in NP and WF cells: HSP > mitochondria, microsomes and lysosomes, HDP > granules. In contrast to As, there were no significant differences in Pb concentration among the subcellular fractions (Supplementary Figure S6), where the HSP and the granule fractions contained lower Pb content (Supplementary Figure S5).

#### 3.2.3. Cerium and Lanthanum

In both fish species, the mitochondria (WF: 23% ± 6%; NP: 24% ± 6%) and the microsomes and lysosomes (WF: 24% ± 9%; NP: 27% ± 9%) contributed the most to the total hepatic Ce burden compared to other subcellular fractions (Figure 4). A similar predominant role of both “sensitive” sites was reported for La, where metal proportions varied from 22% to 32% (Figure 4). In contrast to class B metals, the metal contribution of the HSP fraction in NP and WF livers was not greater than 5% of the total hepatic levels for La and Ce. In both fish species, most of the total intracellular content of La (WF: 59% ± 10%; NP: 61% ± 5%) and Ce (WF: 54% ± 10%; NP: 57% ± 6%) were found in the metal-sensitive compartment (Figure 4). These average values were significantly higher than those reported in the metal-detoxified compartment for La (WF: 18% ± 8%; NP: 19% ± 6%) and for Ce (WF: 20% ± 9%; NP: 20% ± 5%) (Figure 4).

Unlike class B metals, Ce and La concentrations in the microsomes and lysosomes and in the mitochondria fractions were generally higher than in the granules and HSP fractions (Supplementary Figure S6). In WF cells, the mean concentration of La (0.05 ± 0.04 nmol g^−1^) and Ce (0.04 ± 0.03 nmol g^−1^) in the microsomes and lysosomes was more than 10 times higher than in the HSP fractions. In addition, the microsome and lysosome fractions in NP cells exceeded the metal concentration of this cytosolic fraction by a ratio of 4.5-fold and 6.5-fold for Ce and La, respectively (Supplementary Figure S6).

#### 3.2.4. Differences in the Subcellular Partitioning Among Trace Elements

**Figure 5:**
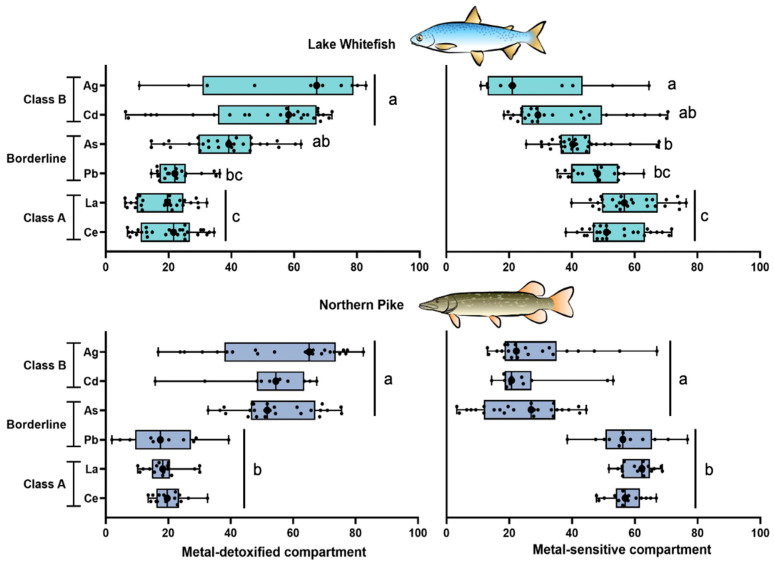
Box and whisker distribution of the metal contribution (%; n = 11–29) of metals belonging to different classes (Class B, Borderline, A) in the metal-detoxified compartment and in the metal-sensitive compartment of the liver of lake whitefish (*Coregonus clupeaformis*) and northern pike (*Esox lucius*). Each dot represents individual fish value. Bars with different letters indicate that the differences are significant (Kruskal-Wallis test followed by Dunn’s test using Bonferroni correction, *p* < 0.05).

Our results revealed differences in the subcellular partitioning of TEs from different ecotoxicological classes based on chemical affinities (Figure 5). The liver cells of NP and WF were able to accumulate class B metals and As (a borderline metal) as detoxified forms in a greater proportion (up to 85%) than those observed for both REEs studied (class A). In contrast to this subcellular partitioning pattern for class B metals and As, the liver cells of NP and WF were not able to detoxify more than 40% of the total intracellular La, Ce, and Pb (Figure 5). Correspondingly, more than 35% of the total level of these two REEs and Pb were found in the “sensitive” sites isolated from both fish livers. Compared with REEs, the sensitive sites showed the lowest TE contributions for class B and As in both fish species.

## 4. Discussion

### 4.1. Trace Metal Concentrations in Fish Liver

The hepatic concentrations of TEs found in this study were generally similar to those measured in fish (with similar morphology and age) reported in previous works. TE concentrations in the liver cells of NP (Ag: 0.18–18 nmol g^−1^; As: 3.3–27 nmol g^−1^; Pb: 0.09–0.20 nmol g^−1^) and WF (Ag: 2.3–27 nmol g^−1^; As: 3.3–39 nmol g^−1^; Pb: 0.09–11 nmol g^−1^) captured in the same area were close to those presented here [31]. In addition, As measurements in the liver of NP (14 nmol g^−1^) and WF (5.6 nmol g^−1^) collected previously from Yellowknife Bay did not differ greatly from recent As measurements in Bay 1 and Bay 2 sites [45]. Among the TEs measured in both fish species, As, Cd, Pb, and Ag have been consistently associated with mining operations in the Yellowknife area, mainly those conducted in the Giant Mine [27,28]. Both fish species from the Long Lake (which only receives mining-related TEs from atmospheric deposition) showed the higher As concentrations. The livers of NP and WF captured in Bay 1 contained the higher levels of Cd because of the atmospheric depositions as well as tailing and effluent releases from the Giant Mine chronically received in this ecosystem. These results are consistent with previous studies on biota (invertebrates, fish) collected from this area, where the impact of mining activities on the environmental quality of these ecosystems was negatively related to the proximity of sites to the Giant Mine [31,46].

With regard to REEs, spatial differences were also observed where fish species from Bay 1 were the most contaminated in La and Ce. When exploring similarities, including all our bioaccumulation measurements using PCA analyses, REE differed from the metals historically associated with mining activities, such as As, Pb, Cd, and Ag (Supplementary Figure S7). This observation suggests that the source of such contaminants is more related to a geological contribution than to human-related activities conducted in this region. To the best of our knowledge, this is the first measurement of REE in fish from the Yellowknife area; the data provide important baseline information for potential future mining exploration related to REE in the Northwest Territories. In comparing metal concentrations, we observed that the liver of WF is generally more contaminated than that of NP, which agrees well with studies on fish collected from the Yellowknife area [31,45]. This difference may be related to trophic status and to the feeding behaviors of both fish species [47]. Bottom-feeder fish like WF tend to have higher As concentrations than predatory fish like NP, for which lower total As burden has been reported [45].

From a toxicological point of view, the total metal concentrations found in NP and WF livers are typically lower than those reported, causing sublethal effects. For example, adult whitefish *Coregonus lavaretus* captured from the most mining-impacted sites in the Inari-Pasvik watercourse (Finland) showed higher hepatic Cd concentrations (around 20 nmol g^−1^) than those found in WF in this study (maximum at 6 nmol g^−1^) [48]. The authors associated this metal concentration with a decrease in the somatic growth of this fish species. Higher concentrations in the hepatocytes of American eels, *A. rostrata* (ranging from 14 to 20 nmol g^−1^), which have experienced a drastic decline over the last decades, are also greater than the total Ag measurements in NP and WF livers [49]. In addition, histopathological alterations were observed (from grade 1 (mild) to 3 (severe)) in the liver of adult lake whitefish exposed to contaminated food, where the total hepatic As concentrations measured up to 12 nmol g^−1^ (considering that liver tissues are 75% water) [38]. This value, which also resulted in a reduction of WF growth rates, is lower than the As concentrations found in the liver of NP (20 nmol g^−1^) and WF (43 nmol g^−1^), which indicates that the As level measured in this study may pose a health threat to these species.

Furthermore, maximum whole-body concentrations (transforming ww into dw) for La (0.91 nmol g^−1^) and Ce (1.69 nmol g^−1^) in ten freshwater fish species captured from a reservoir in Washington (US) are relatively similar to those observed in NP and WF from the Yellowknife area [50]. Recently, hepatic concentrations of La (0.22–0.63 nmol g^−1^) and Ce (0.29–0.76 nmol g^−1^) were reported in different fish species collected from the St Lawrence River (Canada), which are similar to our REE measurements [51]. In a study conducted to establish background REE levels in a subarctic environment (Kobbefjord, Greenland), where potential REE mining activity will be developed in the coming years, La and Ce concentrations in the liver of adult arctic char (*Salvelinus alpinus*) were also in the same concentration ranges as those observed in this study [52]. Although both Yellowknife-captured fish species showed lower TE concentrations in the liver, measurements of enzymatic biomarkers related to proteins, lipids, and carbohydrate metabolism, as well as to oxidative stress (e.g., production of reactive oxygen species, activity of antioxidant enzymes) in these animal models, are warranted to assess the risk of such bioaccumulated contaminants [49,53]. Such analyses would provide new insights into the molecular mechanisms by which TEs found in the area affect metal-exposed fish populations in the mining sites.

### 4.2. Subcellular Partitioning of Studied Metals

The subcellular metal partitioning approach has been widely applied recently to address several questions regarding metal detoxification and, more importantly, metal toxicity in aquatic organisms. We applied this approach using an enzymatically validated protocol to deal with key questions regarding the potential risk that trace metals measured in the Yellowknife area posed to both fish species studied. In addition, the subcellular partitioning of Ce and La (as REE) in field-collected aquatic organisms has rarely been studied, and this study is the first to provide data about the behavior of members of this relevant group of contaminants in metal-exposed fish from mining-contaminated ecosystems.

#### 4.2.1. Class B Elements

Despite the differences in the range of total hepatic concentrations of Ag and Cd, the subcellular partitioning of these elements was consistently similar in both fish species. These two class B metals were found in greater proportions (ranging from 47% to 51%, including all measurements) in the HSP fraction of the hepatic cells of both WF and NP compared to the other subcellular fractions. Our results are consistent with previous subcellular partitioning studies on various fish species (*Anguilla anguilla* and *Anguilla rostrata*, *Catostomus commersonii*, *Perca flavescens*, *Sebastes ruberrimus*), where Cd and Ag were also largely accumulated in the thermostable cytosolic fraction [15,19,21,43]. The binding of both metals in the granule fractions was also reported, which seems to indicate the involvement of this fraction in metal detoxification but is somewhat limited compared to the predominant role of the HSP fraction in accumulating intracellular Cd and Ag. Knowing the HSP fraction contains heat-stable peptides and proteins, including metallothioneins (MT) or metallothionein-like proteins (MTLP) and glutathione (GSH), it suggests that Cd and Ag were predominately bound to these metal chelators (e.g., MT or MTLP, GSH) as detoxified forms in the liver cells of WF and NP. Metallothioneins, as cysteine-rich, heat-stable, low-molecular-weight proteins, play an essential role in metal detoxification in living cells [54,55]. This cytosolic protein could easily interact with both class B metals (also known as soft metals) due to their high affinity for the sulfhydryl groups available on the MT cysteine residues and the overexpressed MT levels (caused by the increasing TE exposure) [4,6]. Considering that Cd and Ag are also inducers of the MT biosynthesis, the metal binding to MT or MTLP could become more efficient with increasing metal exposure, leading to a higher accumulation in this subcellular fraction. Within the HSP fraction, the biochemical interaction of MT and Cd or Ag suggested here for two species of fish, WF and NP, agrees well with the analyses of Cd- and Ag-MT complexes isolated from yellow perch HSP fractions as measured by an on-line SEC-ICP-MS approach [56]. The authors demonstrated that cytosolic Cd and Ag are largely present as MT or MTLP complexes in the liver cells of mining-impacted yellow perch.

Despite the accumulation observed as detoxified forms, the binding of Ag and Cd in the metal-sensitive fractions was also found for both fish liver cells. Nearly all the Ag and Cd accumulated in these sensitive sites were found in the HDP and the mitochondria fractions in WF and in NP cells, respectively. According to Mason and Jenkins [10], metal accumulations in these “sensitive” fractions, where nonessential metals can harmfully interact with physiologically important biomolecules, could lead to adverse effects. Comparing metal accumulation between both compartments, higher proportions of Ag and Cd were measured in the detoxified sites than in the sensitive fractions in WF and NP, which suggests that total hepatic concentrations of Cd and Ag do not represent a concern for the health of these fish species. This observation is consistent with the lower total hepatic Cd and Ag concentrations observed in WF and NP from the Yellowknife area compared to fish from other metal-contaminated sites [48,57].

#### 4.2.2. Borderline Elements

The bioaccumulation range of As and Pb differed between WF and NP, where the maximum total concentration of As and Sb in WF was 2.6-fold and 2.3-fold greater than those for NP, respectively. High percentages of As were measured in the HSP fraction of both fish species (WF: 34%; NP: 54%), which likely contained MT or MTLP and GSH as metal-chelating agents able to neutralize and immobilize trace metals. This predominant role of the HSP fraction implies that As could interact with thiol-containing cytosolic ligands involved in metal detoxification. Studies on Atlantic eels also found that As was largely associated with this cytosolic heat-stable fraction, in which As concentrations increased as well with the increasing total hepatic As concentrations [43]. Significant relationships between As concentrations in the HSP fraction and the total As concentrations in the livers of *Catostomus commersonii* and *Sebastes ruberrimus* were reported [19,21]. With regard to the binding of As and MT or MTPL, this metalloid, in particular the As^+3^ redox state, has been classified as a borderline metal with a relatively low affinity for thiol groups compared to class B metals [4]. However, As^+3^ is not the only As species that could interact with MT or MTLP. Indeed, other studies have found monomethylarsonous acid (MMA) and dimethylarsinous acid (DMA) were also bound to MT [58]. DMA was the predominant As form measured (around 73%) in the livers of NP, whereas other organic arsenic species (OAS), but not DMA or MMA, accounted for 70% of the total As determined in the liver of WF [45]. Consistent with a previous study performed in the Yellowknife area [45], the difference reported between both fish species in As speciation could be associated with the greater accumulation observed in the HSP fraction of NP, where there are more possibilities of As species to interact with MT or MTPL than the limited As forms found in the liver of WF. More chemical and biochemical studies, including the emerging application of LC-based metallomics techniques, are required for further understanding the way in which As species interact within cells, specifically with biomolecules involved in the detoxification or the toxicity of such elements [59].

In contrast to As, Pb was not predominantly found in the fractions involved in metal detoxification. This could be partly related to the lower levels of Pb than As in the liver, which may not trigger a detoxification response. In both fish species, the Pb proportions (as average) observed in any of these detoxification fractions (the HSP or the granule fraction) did not exceed 38% of the total hepatic Pb measured. In both fish species, the main Pb detoxified forms were found in the granule fraction, which may be incorporated into type B granules. Further studies using hyphenated techniques must be carried out to elucidate the interaction of Pb in these subcellular fractions. Despite the differences observed between both borderline metals, the detoxification strategies did not completely neutralize or immobilize either element. Metal accumulation in the putative sensitive fractions was consistently found along the bioaccumulation gradients of both metals. Among these fractions, the mitochondria and the microsomes and lysosomes fractions were the most important sensitive targets of As and Pb in the studied liver cells. For Pb, the total metal proportions found in these fractions were significantly greater to those observed for detoxified forms in NP and WF, which could be an indication of metal toxicity.

#### 4.2.3. Class A Elements

The maximum total hepatic concentration of La and Ce observed in WF was 10-fold higher than that measured in NP. Despite such differences, the subcellular partitioning results of both REE members are very similar between both fish species. Subcellular partitioning results of members of the REE group using validated protocols in field-collected fish are scarce. Some fish studies have been conducted under laboratory-controlled conditions [60] or collected from sites merely impacted by metals [23]. In the present work, we provided subcellular measurements of La and Ce in field-collected and mining-exposed fish. Our results showed that in liver cells of WF and NP, the accumulations of both REE in the granule fraction were consistently higher than that observed in the HSP fraction, which suggests that most of the intracellular detoxified forms of La and Ce were found in metal-rich structures. These results are similar to those obtained in yellow perch from the St. Lawrence River for another element often included as a rare earth, yttrium (Y), where the granule-like fraction was consistently the main detoxification strategy to cope with intracellular Y [23]. Although the role of these mineral inclusions in metal detoxification has been reported more frequently in invertebrates [61], they are also found in vertebrates as lipofuscin granules or metal-rich depositions [62,63]. In this regard, intracellular La was largely associated with “granular precipitates” in rat liver after La chloride exposure using advanced microscopy-relative techniques [64]. However, more research is warranted to confirm if the extrapolation of this in vivo observation is suitable for field-collected aquatic organisms exposed to environmentally realistic La and Ce concentrations in mining areas.

Despite the accumulation of these REE in metal-detoxified compartments in both fish species, metal accumulation of La and Ce in the metal-sensitive compartment was consistently greater than that reported for the detoxified forms. Given that both elements share similar ionic radius, coordination numbers, and ionic charge properties with essential metals, these contaminants could efficiently compete with Ca, Fe, Zn, Mn, and Mg from their binding sites on proteins to form stable complexes, which could result in metal toxicity [65]. This partitioning of rare earth elements towards sensitive fractions in fish liver is in agreement with previous field [23] and laboratory studies on Y [66]. Although we did not measure any toxicity endpoint in WF or NP liver cells, our interpretations agree well with previous studies in which the exposure of living cells to lanthanides, including La or Ce, was associated with free radical imbalances caused by alterations in electron transfer reactions taking place in specific subcellular sites [66]. In hepatic cells of NP and WF, we also observed greater metal accumulation in other sensitive sites such as cytosolic, thermosensible enzymes (located in the HDP fractions) and organelles (pelleted in the microsomes and lysosomes fractions). As class A, both elements have a low probability of interacting with S-containing groups in MTs or MTLPs, and, as a consequence, they are found in larger proportions in the “sensitive” sites, such as cytosolic thermosstable protein fractions. In this specific site, La and Ce would interact with other O-containing ligands, resulting in a decreased or suppressed biological activity of the biomolecules. As reviewed, the biochemical activity of the lanthanides with different intracellular biomolecules is markedly determined by their capacity to mimic essential metals [66]. Because of this chemical behavior, La and Ce (as other members of lanthanide groups) have been associated with blocking Ca-operated channels, replacing essential metals, and inhibiting biological Ca-dependent processes. Comparing the metal-handling strategy of both fish species, we noted that the subcellular partitioning of La and Ce between both compartments in NP cells was very similar to that reported for the WF hepatocytes.

### 4.3. Emerging Classification of Metal Partitioning in Fish Liver

From our results, a general pattern emerged from elemental subcellular partitioning of TEs in different ecotoxicological classes based on chemical affinities. Exploring the different chemical characteristics (i.e., ionization potential, electronegativity, ionic radius, covalent index, ionic index) of measured TEs and the metal contribution in both compartments, significant relationships (*p* < 0.04) were observed for both fish species, but only in the metal-sensitive compartment (MSC) (Supplementary Table S4, Supplementary Figure S8). The results showed that both TE chemical metrics are key parameters to understand and to predict the biochemical behavior of these contaminants within the cells, where they can induce toxic effects once accumulated in this subcellular compartment. Such correlations are consistent with previous studies where QICARs modeling was able to successfully predict the general lethal toxicity of various TE of different aquatic organisms [1,3]. The general patterns presented here for a mining area are generally consistent with another study (using a comparable protocol) on yellow perch in a non-mining area (the St. Lawrence River) where class A (Y, Sr), class B (Cu, Cd, MeHg), and borderline (Fe, Mn) metals were considered [23]. Taken together, these findings strongly suggest that metal classification into class A, B, and borderline can be used as a starting point for predicting metal partitioning between sensitive and detoxified fractions for data-poor metals, including critical elements of emerging ecotoxicological concerns. These correlations should be interpreted with caution because the enzymatic validation of the protocol applied to isolate the mitochondrial fraction indicated that this fraction contained mitochondrial membrane rather than the whole organelle, as expected. In addition to that, our QICAR modelling results were limited to a few characteristics, and therefore further modelling including a wide range of chemical metrics should be necessary for a better explanation of the TE accumulation in sensitive sites.

## 5. Conclusions

In this present work, the total liver concentration as well as the subcellular partitioning of various TEs of class A, B, and borderline groups were determined in the fish liver of Northern pike and Lake whitefish collected from the Yellowknife area. Our results showed that TE concentrations in fish liver were similar to those reported in previous studies, which suggests stable temporal TE contamination in these aquatic organisms. More studies (e.g., using specific toxicity biomarkers) are needed to assess the impact of such bioaccumulated TEs on fish inhabiting this mining-impacted area. In addition, subcellular measurements revealed significant differences between TE classes. While class B elements (Cd, Ag) were largely found as detoxified forms, Ce and La, which were poorly detoxified, accumulated in greater proportions than those of Ag and Cd in the metal-sensitive fractions. Borderline elements (As and Pb) had an intermediate behavior between classes A and B. The covalent index and electronegativity of measured TEs were identified as predictors of the TE contribution in the metal-sensitive compartment by applying QICAR modeling. To our knowledge, this is the first report where the chemical properties of TEs predict the metal accumulation in a specific subcellular fraction, such as mitochondria. These results will help predict TEs of concern (more accumulated in this sensitive site) based on such chemical characteristics in polymetallic-contaminated aquatic organisms and, consequently, establish priorities in further toxicological studies. Such findings are critical for environmental risk assessors in their mission to develop useful protection tools to anticipate the toxicological effects of TEs, specifically those considered as data-poor TEs.

## Data Availability

The original contributions presented in the study are included in the 581 article, further inquiries can be directed to the corresponding author.

## Supplementary Materials

The following supporting information can be downloaded at: https://www.mdpi.com/article/10.3390/toxics13050410/s1. Figure S1. Map of the Yellowknife area and the locations of the 4 sampling sites (Bay 1, Bay 2, Small Lake and Long Lake). Hatched zone indicates the Yellowknife city area and the start symbol the Giant Mine, Figure S2. Proportion of enzymatic activity (means ± SD, %, n = 5) of cytochrome C oxidase (CCO, biomarker of mitochondrial membrane), citrate synthase (CS; biomarkers of mitochondrial matrix) and lactate dehydrogenase (LDH; biomarker of cytosol) in subcellular fractions obtained from the liver of lake whitefish (*C. clupeaformis*, right panel) and northern pike (*E. lucius*, left panel). Bars with different letters indicate that the differences are significant (Kruskal-Wallis test followed by Dunn’s test using Bonferroni correction, *p* < 0.05), Figure S3. Protocol of subcellular partitioning applied to separate livers into subcellular fractions for Northern pike and Lake Whitefish. S: Supernatant; P: pellet, Figure S4. Box and whisker distribution of concentration (nmol g^−1^ dw; n = 10–28) of Ag (upper panels) and Cd (lower panels) in each subcellular fraction and compartment of the liver of the lake whitefish (*Coregonus clupeaformis*) and northern pike *(Esox lucius)*. Each dot represents an individual fish value. Bars with different letters indicate that the differences are significant (lowercase letters for subcellular fractions: Kruskal-Wallis test followed by Dunn’s test using Bonferroni correction; capitalized letters for compartments: Wilcoxon–Mann–Whitney test, *p* < 0.05). MIT: mitochondria; ML: microsomes and lysosomes; HDP: heat-denatured proteins; GRAN: granules; HSP: heat-stable proteins and peptides; MSC: metal-sensitive compartment; MDC: metal-detoxified compartment, Figure S5. Box and whisker distribution of the concentration (nmol g^−1^ dw; n = 19–29) of As (upper panels) and Pb (lower panels) in each subcellular fraction and compartment of the liver of the lake whitefish (*Coregonus clupeaformis*) and northern pike *(Esox lucius)*. Each dot represents an individual fish value. Bars with different letters indicate that the differences are significant (lowercase letters for subcellular fractions: Kruskal-Wallis test followed by Dunn’s test using Bonferroni correction; capitalized letters for compartments: Wilcoxon–Mann–Whitney test, *p* < 0.05). MIT: mitochondria; ML: microsomes and lysosomes; HDP: heat-denatured proteins; GRAN: granules; HSP: heat-stable proteins and peptides; MSC: metal-sensitive compartment; MDC: metal-detoxified compartment. Figure S6. Box and whisker distribution of the concentration (nmol g^−1^ dw; n = 16–26) of La (upper panels) and Ce (lower panels) in each subcellular fraction and compartment of the liver of the lake whitefish (*Coregonus clupeaformis*) and northern pike *(Esox lucius)*. Each dot represents an individual fish value. Bars with different letters indicate that the differences are significant (lowercase letters for subcellular fractions: Kruskal-Wallis test followed by Dunn’s test using Bonferroni correction; capitalized letters for compartments: Wilcoxon–Mann–Whitney test, *p* < 0.05). MIT: mitochondria; ML: microsomes and lysosomes; HDP: heat-denatured proteins; GRAN: granules; HSP: heat-stable proteins and peptides; MSC: metal-sensitive compartment; MDC: metal-detoxified compartment. Figure S7. Principal Component Analysis (PCA) based on total trace metal concentrations in liver cells of lake whitefish (*Coregonus clupeaformis*) and northern pike (*Esox lucius*), Figure S8. Relationship between electronegativity (upper panels) and covalent index (lower panels) with the trace metal contributions (%) in the metal-sensitive compartments (MSCs) of the liver of lake whitefish (*Coregonus clupeaformis,* left panels) and northern pike (*Esox lucius,* right panels). Equation, coefficient of determination (R^2^) and p value (*p*) are given, Table S1. Recoveries (mean values ± standard deviation, n) of certified reference materials used in the trace metal measurements, Table S2. Mass balance recoveries (mean values ± standard deviation, n) calculated for each trace metal in both fish species, Table S3. Ranges (minimum-maximum) of trace metal concentrations (nmol. g^−1^ dw) and bioaccumulation ratios ([M]_max_/[M]_min_) in the liver of both fish species. Ranges of age (years), length (mm), and weight (g) of each fish species is also given. Table S4. Statistical parameters (R^2^; *p*) of the relationship between chemical properties and index with the metal contributions (%) in the metal-sensitive compartment (MSC) as well as in the metal-detoxified compartment (MDC) of the liver of lake whitefish (*Coregonus clupeaformis*) and northern pike (*Esox lucius*). Parameters in bold are significant.
