# Supplementary material for: Subcellular Partitioning of Trace Elements Is Related to Metal Ecotoxicological Classes in Livers of Fish (Esox lucius; Coregonus clupeaformis) from the Yellowknife Area (Northwest Territories, Canada)
Source: Toxics. 2025 May 19;13(5):410. doi: 10.3390/toxics13050410 (PMC12115823; doi:10.3390/toxics13050410)
Supplement: Supplementary file 1 [file toxics-13-00410-s001.zip › toxics-3598239-supplementary.pdf]

Supplementary Material

Subcellular partitioning of trace elements is related to metal ecotoxicological classes in liver of fish (*Esox Lucius; Coregonus clupeaformis*) from the Yellowknife area, Northwest Territories, Canada

Aymeric Rolland[1], Mike Palmer[2], John Chételat[3], Marc Amyot[4], and Maikel Rosabal[1*]

1 Groupe de Recherche Interuniversitaire en Limnologie (GRIL), Département des sciences biologiques, Université du Québec à Montréal (UQAM), Montréal H2X 1Y4, QC, Canada; rosabal.maikel@uqam.ca

2 North Slave Research Centre, Aurora Research Institute, Aurora College, Yellowknife, NT, Canada; mpalmer@auroracollege.ca

3 Environment and Climate Change Canada, National Wildlife Research Centre, Ottawa K1S 5B6, ON, Canada; john.chetelat@ec.gc.ca

4 Groupe de Recherche Interuniversitaire en Limnologie (GRIL), Département de sciences biologiques, Université de Montréal, Montréal H2V 0B3, QC, Canada; m.amyot@umontreal.ca

* Correspondence author: rosabal.maikel@uqam.ca

Table S1. Recoveries (mean values ± standard deviation, n) of certified reference materials used in the trace metal measurements.

Trace metals	TORT-2[a] (%, n = 3)	DOLT-5[b] (%, n = 3)	BCR-668[c] (%, n = 6)
Ag		81 ± 4	
Cd	102 ± 2	95 ± 3	
As	103 ± 2	100 ± 2	
Pb	126 ± 4		
La			101± 4
Ce			99 ± 5

[a] lobster hepatopancreas (National Research Council of Canada, NRCC, Ottawa, ON, Canada)
[b] dogfish liver (National Research Council of Canada, NRCC, Ottawa, ON, Canada)
[c] mussel tissue (Institute for Reference Materials and Measurements, IRMM, Geel, Belgium)

Table S2. Mass balance recoveries (mean values ± standard deviation, n) calculated for each trace metal in both fish species.

Trace metals	Recovery mean (%) *E. lucius*	Recovery Mean (%) *C. clupeaformis*
Ag	108 ± 18, n = 23	100 ± 18, n = 10
Cd	105 ± 14, n = 11	93 ± 12, n = 28
As	84 ± 10, n = 25	87 ±11, n = 29
Pb	95 ± 120, n = 15	106 ± 16, n = 19
La	98 ± 21, n = 17	105 ± 14, n = 26
Ce	98 ± 20, n = 16	105 ± 16, n = 26

Table S3. Ranges (minimum-maximum) of trace metal concentrations (nmol. g^{-1} dw) and bioaccumulation ratios ($[M]_{max}$ /$[M]_{min}$) in the liver of both fish species. Ranges of age (years), length (mm), and weight (g) of each fish species is also given.

Variables	Trace metals					
	Ag	Cd	As	Pb	La	Ce
Esox lucius						
[M] range (nmol g^{-1} dw)	0.96 – 16.0	0.12 – 6.48	1.81 – 18.8	0.14 – 1.91	0.01 – 0.17	0.02 – 0.29
[M]max /[M]min	17	54	10	13	14	15
Age range (years)	4 – 16					
Length range (mm)	4 – 744					
Weight range (g)	680 – 3200					
Coregonus clupeaformis						
[M] range (nmol g^{-1} dw)	0.36 – 4.26	0.22 – 6.10	2.39 – 42.1	0.16 – 1.90	0.04 – 0.63	0.06 – 0.83
[M]max /[M]min	12	27	18	11	15	14
Age range (years)	6 – 25					
Length range (mm)	355 – 480					
Weight range (g)	600 – 3000					

Table S4. Statistical parameters (R^2 ; *p*) of the relationship between chemical properties and index with the metal contributions (%) in the metal-sensitive compartment (MSC) as well as in the metal-detoxified compartment (MDC) of the liver of lake whitefish (*Coregonus clupeaformis*) and northern pike (*Esox lucius*). Parameters in bold are significant.

Lake Whitefish

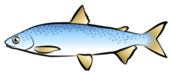

Northern Pike

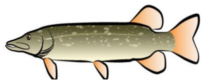

	Lake Whitefish		Northern Pike	
Variables	MSC	MDC	MSC	MDC
Ionization potential	R^2: 0.27<br>*p* = 0.097	R^2: 0.28<br>*p* = 0.80	R^2: 0.03<br>*p* = 0.09	R^2: 0.04<br>*p* = 0.62
Electronegativity	**R^2: 0.59**<br>***p* = 0.01**	R^2: 0.55<br>*p* = 0.54	**R^2: 0.59**<br>***p* = 0.005**	R^2: 0.42<br>*p* = 0.83
Ionic radius	R^2: 0.52<br>*p* = 0.58	R^2: 0.49<br>*p* = 0.07	R^2: 0.09<br>*p* = 0.30	R^2: 0.14<br>*p* = 0.16
Covalent index	**R^2: 0.20**<br>**p = 0.04**	R^2: 0.18<br>*p* = 0.47	**R^2: 0.54**<br>***p* = 0.002**	R^2: 0.30<br>*p* = 0.48
Ionic index	R^2: 0.03<br>*p* = 0.08	R^2: 0.02<br>*p* = 0.14	R^2: 0.09<br>*p* = 0.016	R^2: 0.05<br>*p* = 0.06

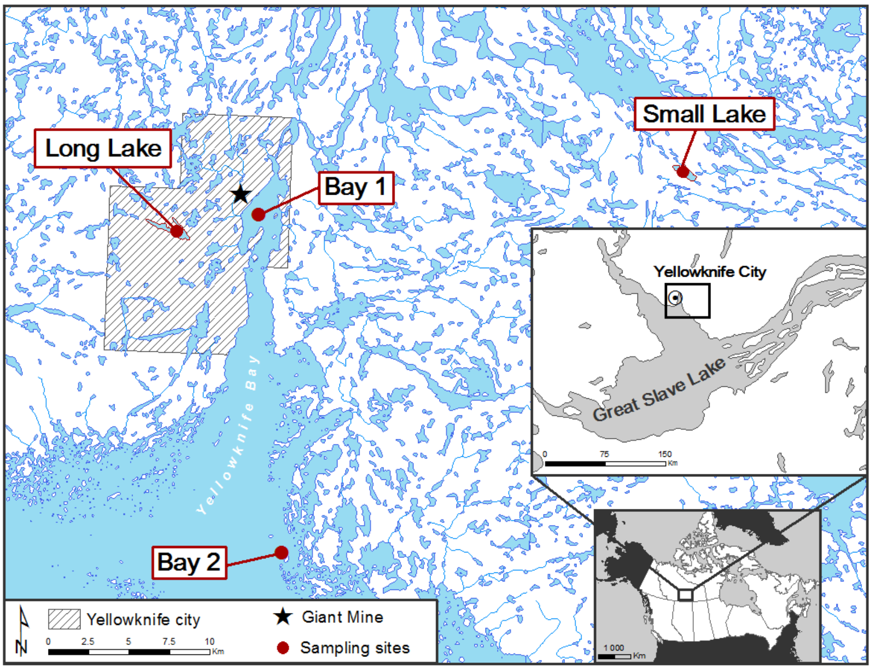

Figure S1. Map of the Yellowknife area and the locations of the 4 sampling sites (Bay 1, Bay 2, Small Lake and Long Lake). Hatched zone indicates the Yellowknife city area and the start symbol the Giant Mine.

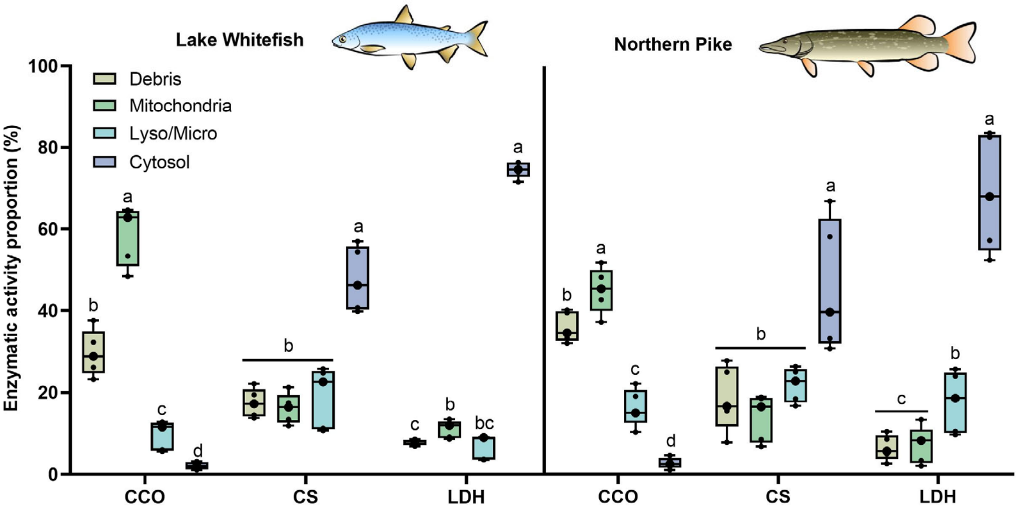

Figure S2. Proportion of enzymatic activity (means ± SD, %, n = 5) of cytochrome C oxidase (CCO, biomarker of mitochondrial membrane), citrate synthase (CS; biomarkers of mitochondrial matrix) and lactate dehydrogenase (LDH; biomarker of cytosol) in subcellular fractions obtained from the liver of lake whitefish (*C. clupeaformis*, right panel) and northern pike (*E. lucius*, left panel). Bars with different letters indicate that the differences are significant (Kruskal-Wallis test followed by Dunn’s test using Bonferroni correction, $p < 0.05$).

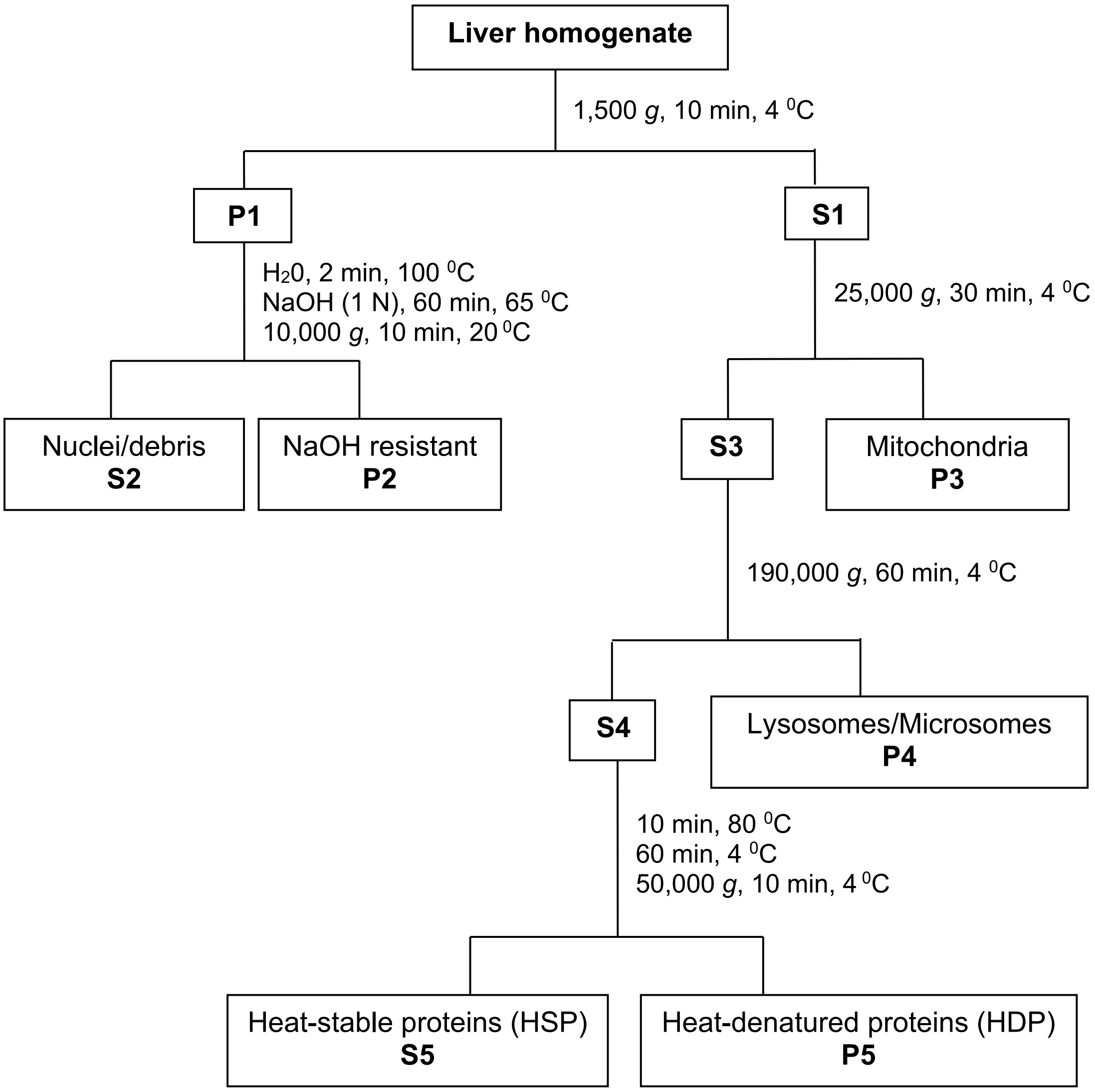

Figure S3. Protocol of subcellular partitioning applied to separate livers into subcellular fractions for Northern pike and Lake Whitefish. S: Supernatant; P: pellet

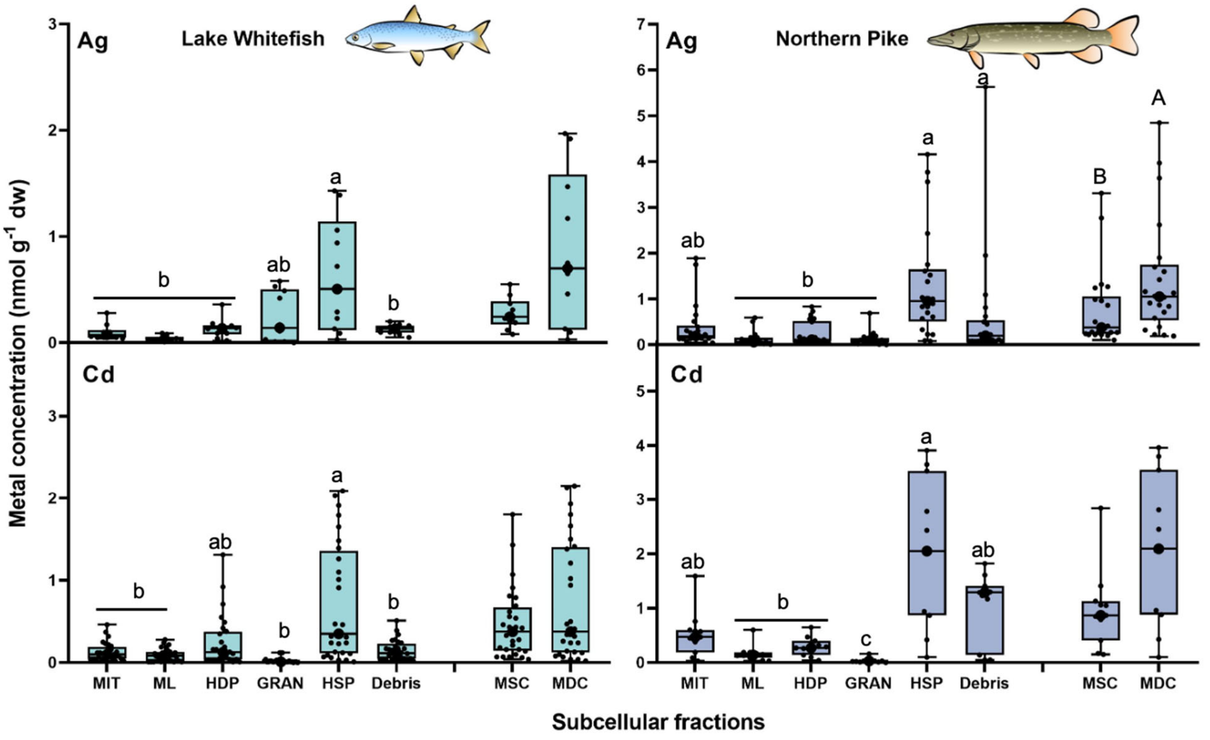

Figure S4. Box and whisker distribution of concentration (nmol g^{-1} dw; n = 10 – 28) of Ag (upper panels) and Cd (lower panels) in each subcellular fraction and compartment of the liver of the lake whitefish (*Coregonus clupeaformis*) and northern pike *(Esox lucius)*. Each dot represents an individual fish value. Bars with different letters indicate that the differences are significant (lowercase letters for subcellular fractions: Kruskal-Wallis test followed by Dunn's test using Bonferroni correction; capitalized letters for compartments: Wilcoxon–Mann–Whitney test, $p <$ 0.05). MIT: mitochondria; ML: microsomes and lysosomes; HDP: heat-denatured proteins; GRAN: granules; HSP: heat-stable proteins and peptides; MSC: metal-sensitive compartment; MDC: metal-detoxified compartment.

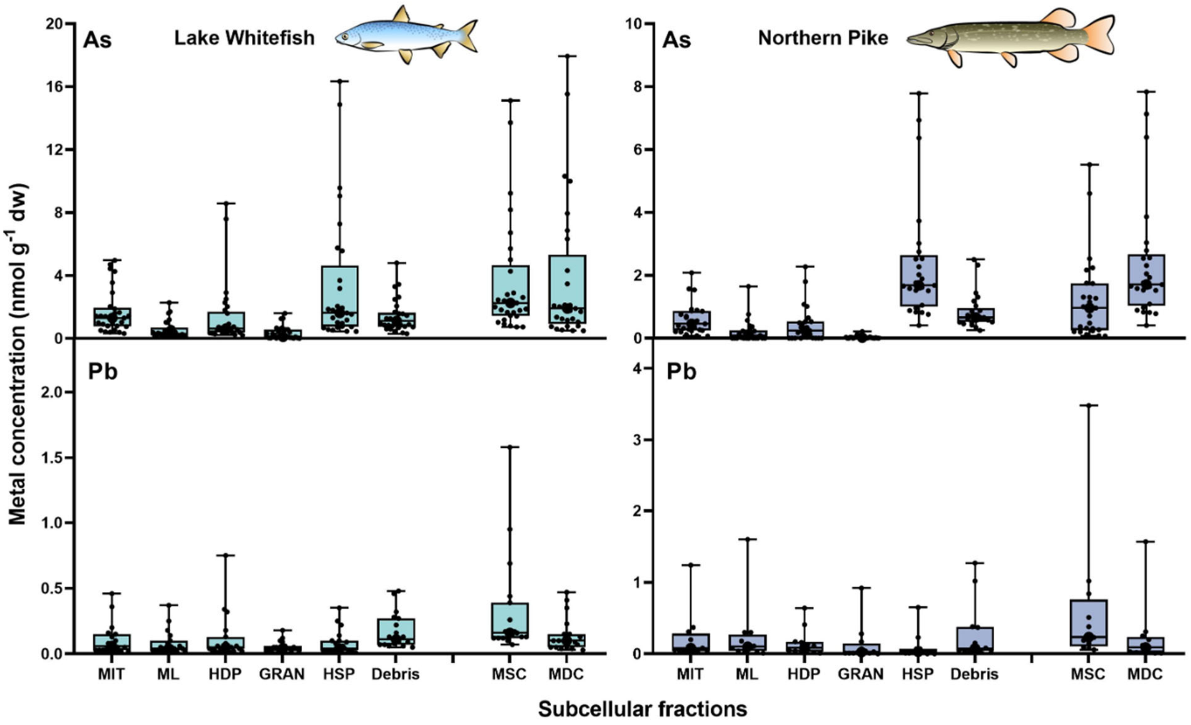

Figure S5. Box and whisker distribution of the concentration (nmol g^{-1} dw; n = 19 – 29) of As (upper panels) and Pb (lower panels) in each subcellular fraction and compartment of the liver of the lake whitefish (*Coregonus clupeaformis*) and northern pike *(Esox lucius)*. Each dot represents an individual fish value. Bars with different letters indicate that the differences are significant (lowercase letters for subcellular fractions: Kruskal-Wallis test followed by Dunn's test using Bonferroni correction; capitalized letters for compartments: Wilcoxon–Mann–Whitney test, $p < 0.05$). MIT: mitochondria; ML: microsomes and lysosomes; HDP: heat-denatured proteins; GRAN: granules; HSP: heat-stable proteins and peptides; MSC: metal-sensitive compartment; MDC: metal-detoxified compartment.

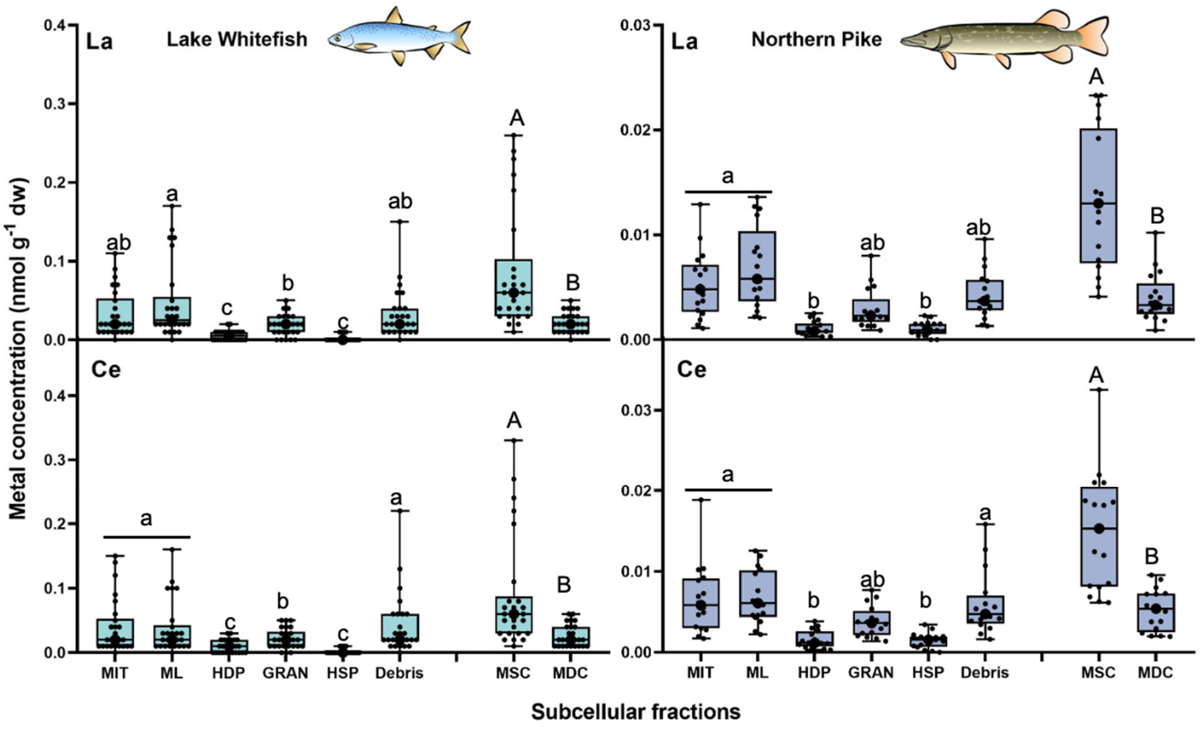

Figure S6. Box and whisker distribution of the concentration (nmol g^{-1} dw; n = 16 – 26) of La (upper panels) and Ce (lower panels) in each subcellular fraction and compartment of the liver of the lake whitefish (*Coregonus clupeaformis*) and northern pike *(Esox lucius)*. Each dot represents an individual fish value. Bars with different letters indicate that the differences are significant (lowercase letters for subcellular fractions: Kruskal-Wallis test followed by Dunn's test using Bonferroni correction; capitalized letters for compartments: Wilcoxon–Mann–Whitney test, $p < 0.05$). MIT: mitochondria; ML: microsomes and lysosomes; HDP: heat-denatured proteins; GRAN: granules; HSP: heat-stable proteins and peptides; MSC: metal-sensitive compartment; MDC: metal-detoxified compartment.

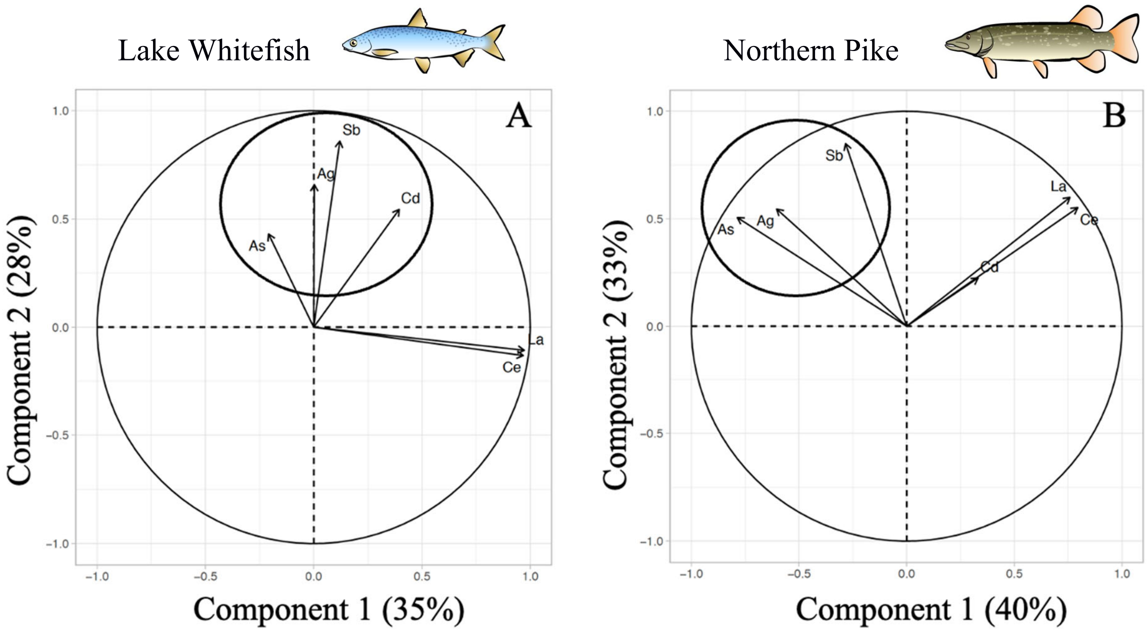

Figure S7. Principal Component Analysis (PCA) based on total trace metal concentrations in liver cells of lake whitefish (*Coregonus clupeaformis*) and northern pike (*Esox lucius*).

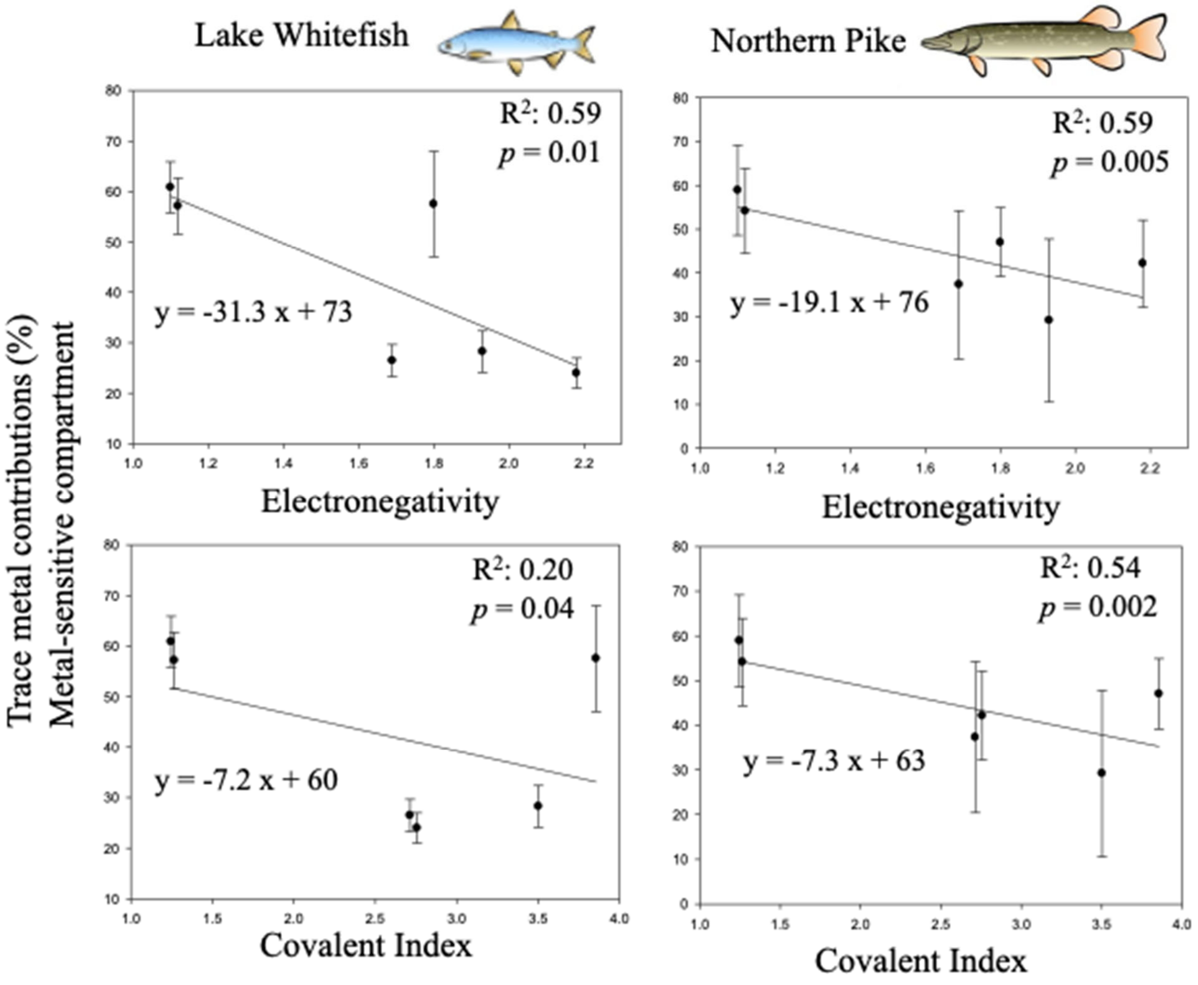

Figure S8. Relationship between electronegativity (upper panels) and covalent index (lower panels) with the trace metal contributions (%) in the metal-sensitive compartments (MSCs) of the liver of lake whitefish (*Coregonus clupeaformis,* left panels) and northern pike (*Esox Lucius,* right panels). Equation, coefficient of determination (R^2) and p value (p) are given.
